# Does Statin or ASA Affect Survival When Prostate Cancer Is Treated with External Beam Radiation Therapy?

**DOI:** 10.1155/2014/184297

**Published:** 2014-03-03

**Authors:** J. Caon, M. Paquette, J. Hamm, T. Pickles

**Affiliations:** ^1^BC Cancer Agency Radiation Therapy Program, BC Cancer Agency, Vancouver Centre, 600 West 10th Avenue, Vancouver, BC, Canada V5Z 4E6; ^2^University of British Columbia, 2329 West Mall, Vancouver, BC, Canada V6T 1Z4; ^3^Cancer Surveillance & Outcomes, Population Oncology, BC Cancer Agency, 600 West 10th Avenue, Vancouver, BC, Canada V5Z 4E6

## Abstract

*Background*. Prior studies evaluating the effect of statins or acetylsalicylic acid (ASA) on the survival of men receiving prostate cancer were treatment have reported conflicting results, and have not adjusted for comorbidity. Our aim is to investigate the influence of statins and ASA on prostate cancer survival, when comorbidity is adjusted for, in men treated with external beam radiation therapy (EBRT) for prostate cancer. *Methods*. A cohort of 3851 patients with prostate cancer treated with curative EBRT ± androgen deprivation therapy (ADT) between 2000 and 2007. Stage, treatment, medication use, and Charlson comorbidity index (CCI) scores were analyzed. *Results*. Median followup was 8.4 years. Mean age was 70.3 years. Neoadjuvant ADT was used in 67%. Statins were used in 23%, ASA in 24%, and both in 11%. Comorbidity scores were 0 in 65%, 1 in 25%, and ≥2 in 10% of patients. Statin and ASA use were associated with increased age and comorbidity. Although statin and ASA use were significantly associated with improved prostate cancer specific survival (PCSS) on univariate analysis, neither were on multivariate analysis. *Conclusion*. Neither statin nor ASA use impacted PCSS on multivariate competing risks analysis. Survival was impacted by increased comorbidity as well as statin and ASA use.

## 1. Introduction

As a medication class, 3-hydroxy-3-methyl-glutaryl-CoA reductase inhibitors, commonly known as statins, are prescribed to lower cholesterol and reduce the risk of death from cardiovascular disease [1]. However, statins may also modulate prostate cancer through alteration in the cholesterol levels required for signal transduction of prostate cancer cells [2, 3]. Statins may reduce androgen receptor expression and activity which could lead to reduced prostate cancer cell proliferation and increased apoptosis [4, 5] and a reduction in prostate specific antigen (PSA) release by such cells [4]. A number of other characteristics important to a neoplastic cell may also be altered in the presence of statin medications [6].

Acetylsalicylic acid (ASA) is an antiplatelet agent [7]. The effect on cancer is felt to be related to its properties as a nonsteroidal anti-inflammatory, particularly its effect on COX-2 receptors [8]. There is also a complex interplay between blood coagulation and cancer [9].

Studies evaluating the effect of statins or ASA on outcomes of men receiving curative intent treatment for prostate cancer have reported conflicting results. Acetylsalicylic acid has been associated with a decreased risk of prostate cancer [10]. In men with a preexisting diagnosis of prostate cancer, a prospective study did not show an association between this medication and prostate cancer death [11]; this is in contrast to a more recent study which found a lower risk of prostate cancer death in users of acetylsalicylic acid [12]. Some reports have suggested that statin use was associated with improved biochemical control [13, 14] but its effect in survival is less clear [13, 15, 16].

While differences in the health of users and nonusers of certain medications, such as statins, may impact survival outcomes [17], data accounting for comorbidity among patients with prostate cancer remains lacking. In 2010, an editorial by D'Amico [17] advocated for the inclusion of comorbidity in studies assessing the impact of such medications. This study investigates associations between prostate cancer survival and statin and/or ASA use in men treated with curative intent radiation therapy, after adjusting for comorbidity.

## 2. Methods and Materials

A retrospective electronic chart review was conducted of patients with newly diagnosed localized prostate cancer treated in British Columbia (BC) with curative intent external beam radiation therapy (EBRT) for prostate cancer from January 1, 2000, to December 31, 2007. The BC Cancer Agency (BCCA) is a provincial cancer care institution that managed five regional centres providing all of the radiation therapy in BC during the study era. The study period was chosen because statin use was relatively uncommon before this time. Data were obtained from electronic linkage to existing data sources, including tumor marker, cancer registry, and death registry records, supplemented by review of the electronic chart for comorbidity and medication use.

Of initial 6144 patients, 2091 were excluded because they were not treated with EBRT. 4053 charts were reviewed and additional 202 patients were excluded because they had nonadenocarcinoma histology, noncurative treatment intent, prior orchiectomy, postprostatectomy radiation, brachytherapy boost, incomplete radiation course, incomplete electronic records, or no information about medication use. The final cohort included 3851 subjects.

Patient characteristics analyzed were age at diagnosis, income level (generated from tables of average income by postal code), and comorbidity using the Charlson comorbidity index (CCI) which was collected retrospectively by review of charts. The CCI is the most widely used tool to measure comorbidity and is valid in predicting mortality risk in a number of conditions, including cancer [18]. Specific comorbid conditions, including myocardial infarction, congestive heart failure, peripheral vascular disease, cerebrovascular disease, dementia, chronic obstructive pulmonary disease, connective tissue disease, peptic ulcer disease, liver disease (mild to severe), diabetes (with or without end organ damage), hemiplegia, renal disease (moderate to severe), malignancy, leukemia, lymphoma, metastatic solid malignancy, and AIDS, are weighted with different scores [19]. As prostate cancer was present in all subjects, this was not included in the Charlson score generated.

Statin and ASA medication use was coded from documentation at the time of initial consultation, which included referring physician notes, consultation reports as well as the patients written list of medication provided at the time of treatment consultation. “Statins” included a range of different types: atorvastatin, rosuvastatin, pravastatin, simvastatin, lovastatin, pitavastatin, fluvastatin, and statin not otherwise specified (NOS). “ASA” included acetylsalicylic acid only. Duration of medication use was not analyzed.

Clinicopathologic characteristics analyzed were stage, Gleason score, and initial prostate specific antigen (iPSA), allowing classification into a National Comprehensive Cancer Network (NCCN) risk group (low, intermediate, and high risk). Treatment characteristics included EBRT dose, adjuvant androgen deprivation therapy (ADT) use, and ADT duration. The year of radiation treatment was stratified at the median (2004).

Prostate cancer specific survival (PCSS) and overall survival (OS) were the primary endpoints of this analysis. Biochemical control and metastasis-free endpoints were not feasible due to nonstandardized followup practices and nonuniform indications for imaging or intervention upon relapse.

Descriptive statistics with cross-tabulations were employed. Analysis of survival endpoints was completed using univariate Kaplan-Meier statistics; univariate and multivariate hazard ratio models using Fine and Gray's competing risks analysis [20]. Data analyses were completed using Statistical Package for Social Sciences version 17.0 (SPSS, Chicago, IL, USA) and the SAS statistical software package (SAS version 9.3; SAS Institute Inc., Cary, NC). Significance was defined as *P* ≤ 0.05. The study was approved by the Research Ethics Board of the University of British Columbia.

## 3. Results

Clinicopathologic characteristics of the entire cohort and according to prostate cancer risk groups are summarized in Table 1. The mean age was 70.3 years (range 45 to 88 years). The mean EBRT dose was 70.9 Gy. Most patients (67%) received neoadjuvant ADT (mean duration 11.9 months). The distribution of prostate cancer risk groups was high risk in 44%, intermediate risk in 40%, and low risk in 14%. Charlson comorbidity scores were 0 in 65%, 1 in 25%, and ≥2 in 10% of subjects. Comorbidity indices were not associated with prostate cancer risk group (*P* = 0.425) or age (*P* = 0.120).

As expected there were significant differences (*P* < 0.001) between statin and ASA users and nonusers in regard to Charlson comorbidity scores (Table 1), with increasing comorbidity being associated with increased use of these medications. Overall, statins were used in 914 (24%) patients. Statin users were older (*P* = 0.013) and had a higher CCI score (*P* < 0.001) and a lower income level (*P* = 0.04). ASA medications were used in 917 (24%) patients. These patients were also older (*P* < 0.001) with higher CCI scores (*P* < 0.001).

**Figure 1:**
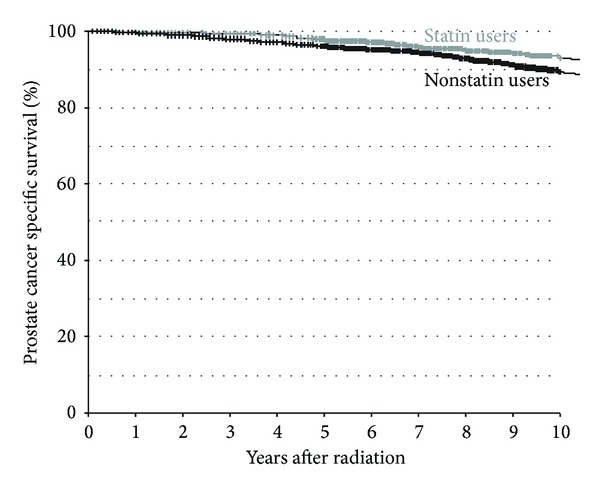
Prostate cancer specific survival between statin users and nonusers.

Median followup for survival was 8.4 years. The association between statin or ASA use with PCSS and OS at 10 years is shown in Table 2. On Fine and Gray competing risks analysis, there was an improvement in PCSS with the use of statins (10-year survival 94.1% versus 91.2%, *P* = 0.031, Figure 1) and with ASA (93.4% versus 91.3%, *P* = 0.004). Neither statin nor ASA use was associated with OS (*P* = 0.83 and 0.37, resp.). As expected, those with higher risk prostate cancer had reduced PCSS and OS (both *P* < 0.001), and men with higher comorbidity had inferior 10-year OS: 67%, 56%, and 47% for CCI score 0, 1, and ≥2, respectively (*P* < 0.001).

The univariate and multivariate hazard ratio model using competing risks is reported for PCSS in Table 3. As expected, lower risk cancers (lower iPSA, T-stage, and Gleason score) were associated with improved PCSS on both univariate and multivariate analysis. However, increased comorbidity, as reflected by CCI score ≥2, had improved PCSS; this is likely due to the influence of competing risks. The improved PCSS seen on univariate analysis in statin and ASA users was not borne out in the multivariate model.

## 4. Discussion

This study is among the first to examine prostate cancer survival outcome relative to both statin and ASA use with comorbidity in a population-based cohort of men with localized prostate cancer treated with curative intent EBRT. When taking comorbidity into account using a multivariate completing risks analysis, neither statin nor ASA use was associated with PCSS.

Population-based studies are typically undertaken to determine whether or not laboratory results are observed at a clinical level. Some studies suggest that statin use is associated with reduced risks of prostate cancer, especially with more aggressive disease [21–23], with larger benefits seen with longer duration [21, 23] and dose [23] of medication, but other studies have not been conclusive in this regard [24–26]. Tagalakis et al. [27] showed that, at a population level, men who used anticoagulants were less likely to be diagnosed with prostate cancer, a decrease in risk that was not seen for any other urogenital cancer and appeared to be related to the duration of medication use. Other studies have also reported a reduced risk of prostate cancer from the use of Warfarin [28] and Aspirin [29] but again this has not been established unanimously [30].

If there is a potential benefit from statins or ASA in lowering the risk of prostate cancer, could the use of these medications affect outcomes in men with known prostate cancer? Several retrospective studies have examined this question with conflicting results. Some investigators [31], but not others [32–34], have reported improved biochemical control among men on statins who underwent radical prostatectomy. Others have observed better biochemical control with statin use in men treated with EBRT [13, 14] and have postulated that this may have been mediated through cholesterol or LDL [13]. Statin use was not associated with biochemical control among patients with prostate cancer treated with brachytherapy, perhaps because of the very high control rates achieved with this modality in comparison with EBRT [33]. Similarly studies, which included ASA with other anticoagulants, found improved biochemical control in prostate cancer patients for patients on such medication [12].

Statin use has been associated with better progression-free survival [16] and lower recurrence risk [15] among men with prostate cancer treated with curative intent on univariate but not multivariate analysis [16]. Gutt et al. [13] found statin use to be associated with better relapse-free survival and freedom from androgen deprivation therapy but not overall or cause-specific survival. The effect ASA may have on prostate cancer outcomes is similarly controversial. In 2012 alone, studies have been published and have found both no association [11] and reduced risk [12] of cancer specific mortalityin men who receive surgery or radiotherapy for prostate neoplasia.

Available studies examining prostate cancer specific and overall survival outcomes in association with statin or ASA use have been limited by a lack of information on subjects' comorbidity. The current study, which used the Charlson comorbidity index, a standardized and validated tool to examine comorbidity, demonstrated that men with a lower CCI had a lower risk of death compared to men with a higher CCI. Statin and ASA medication was used more commonly among men with higher CCI, particularly cardiovascular conditions, warranting the prescription of these medications.

There are several limitations of this study. It is a retrospective review and therefore the reporting of data, including medication use and comorbidities, may not have been complete. Medication use was obtained from the patient's written response to a question at the time of clinic admission and was then transcribed into the consultation record and may be subject to potential inaccuracy in reporting. Medication duration (prior and subsequent to treatment) and compliance was unknown. Our sample size, although large, might not have been enough to detect small difference in PCSS in those taking statins or ASA. Endpoints such as biochemical failure or metastasis-free survival were not reliably available. Death information was obtained from automated lists from BC Vital Statistics Agency, and annual updates from the Canadian national death registry are made. There is little outmigration from BC, but some men's vital statistics may be missing if they emigrated from BC or Canada. Despite these limitations, the current study's findings are of value in documenting the frequency of statin and ASA use among patients with localized prostate cancer and the associated survival outcomes stratified by comorbidity. These findings may be used to inform study design and sample estimates for future trials examining the use of these medications in the setting of prostate cancer [35].

## 5. Conclusion

Statin and ASA use were associated with improved PCSS on univariate analysis but not on multivariate analysis when assessed for competing risks. As expected, statin and ASA are used more often with increased comorbidity, which is significantly associated with worse survival.

Prospective evaluation of the impact of statin use in men with prostate cancer treated with radiation therapy is warranted because of the potential for reducing the mortality of prostate cancer, which remains the 3rd commonest cause of cancer death in the Western world.

## Figures and Tables

**Table 1:** Patient, tumor, and treatment characteristics in the entire cohort and according to use of statin and ASA.

Characteristics	Entire cohort (*N* = 3851)	No statin/ASA (*n* = 2428)	Statin, no ASA (*n* = 506)	ASA, no statin (*n* = 509)	Statin + ASA (*n* = 408)
Median age	71 yrs	71 yrs	71 yrs	72 yrs	72 yrs

Charlson comorbidity index					
0	65% (2507)	73% (1773)	51% (260)	62% (313)	40% (161)
1	25% (961)	20% (473)	33% (166)	29% (145)	43% (177)
≥2	10% (383)	7.5% (182)	16% (80)	10% (51)	17% (70)

Low risk	14% (563)	14% (334)	15% (80)	14% (72)	17% (69)

Intermediate risk	39% (1534)	37% (900)	40% (201)	46% (236)	45% (182)

High risk	44% (1718)	47% (1141)	33% (217)	38% (191)	36% (146)

Null risk	2% (83)	2% (53)	2% (8)	2% (10)	2% (11)

Radiation dose Gy ^a^Median (range)	70 Gy (50–80 Gy)	70 Gy (52.50–78 Gy)	70 Gy (52.50–78 Gy)	70 Gy (52.50–78 Gy)	70 Gy (52.50–78 Gy)

Radiation dose BED_1.5_ ^a^Median (range)	163 (126–201)	163 (126–201)	163 (133–201)	163 (128–195)	163 (140–182)

Frequency ADT^b^ use	67%	71%	61%	63%	59%

Mean duration ADT² (if used)	16 mo	18 mo	18 mo	18 mo	1 mo

^a^RT: radiation therapy; ^b^ADT: androgen deprivation therapy.

**Table:** (a) Prostate cancer specific survival (PCSS) calculated using competing risk analysis by Fine and Gray

	Treatment	No treatment	*P* value
Statin	94.1%	91.2%	0.031
ASA	93.4%	91.3%	0.004

**Table:** (b) Overall survival, Kaplan-Meier log rank analysis

	Treatment	No treatment	*P* value
Statin	63.1%	62.3%	0.827
ASA	61.8%	62.8%	0.371

**Table 3:** Multivariate model for competing risk analysis using Fine and Gray's test [20] of significance of prostate cancer specific survival.

Variable	Univariate analysis				Multivariate analysis^a^		
	Hazard ratio	95% confidence interval	*P* value	10 yr PCSS %	Hazard ratio	95% confidence interval	*P* value
Statin (yes versus no)	0.656	0.478–0.898	0.0086	94.1 versus 91.1	0.769	0.548–1.08	NS

ASA (yes versus no)	0.728	0.537–0.985	0.040	93.4 versus 91.3	0.911	0.648–1.282	NS

Age	0.986	0.968–1.005	0.14	—	0.988	0.972–1.005	NS

Year of treatment (≥2004 versus ≤2003)	1.176	0.924–1.498	0.19	95.9 versus 98.5^b^	1.172	0.897–1.530	NS

Radiation dose	1.000	0.999–1.000	0.13	—	1.000	0.999–1.000	NS

ADT^c^ (yes versus no)	1.789	1.418–2.500	0.0003	90.5 versus 94.5	0.710	0.481–1.049	0.085

iPSA^d^	2.764	1.988–3.843	<0.0001	—	1.59	1.13–2.24	0.0085

*T* stage				97.1			
(2 versus 1)	2.419	1.532–3.820	0.0002	93.5	2.10	1.33–3.33	0.0015
(3 versus 1)	5.916	3.766–9.924	<0.0001	85.6	3.83	2.41–6.08	<0.0001
(4 versus 1)	18.682	10.17–34.31	<0.0001	64.1	11.38	6.11–21.21	<0.0001

Charlson index				90.9			
(1 versus 0)	0.749	0.563–0.998	0.048	92.8	0.76	0.57–1.02	0.072
(≥2 versus 0)	0.495	0.298–0.823	0.0067	95.1	0.53	0.32–0.87	0.013

Gleason score				96.7			
(7 versus ≤6)	2.611	1.823–3.739	<0.0001	92.0	1.94	1.34–2.82	0.005
(≥8 versus ≤6)	5.718	3.987–8.202	<0.0001	83.2	3.21	2.16–4.78	<0.0001

NS: not significant (*P* > 0.05).

^a^Variables with *P*  value ≤ 0.3 in univariate analysis were included in model for multivariate analysis.

^b^Year of treatment is 5-year survival.

^c^iPSA: initial pretreatment PSA.

^d^ADT: androgen deprivation therapy.
